# Editorial: Theory of mind

**DOI:** 10.3389/fpsyg.2024.1370048

**Published:** 2024-02-09

**Authors:** Alfonsina D'Iorio, Chiara Baiano, Maria Dolores Roldan-Tapia, Gabriella Santangelo

**Affiliations:** ^1^Department of Psychology, University of Campania Luigi Vanvitelli, Caserta, Italy; ^2^Department of Psychology, University of Almería, Almería, Spain

**Keywords:** theory of mind, social cognition, social functioning, joint action, cognitive rehabilitation

## Introduction

Social cognition is defined as the processing of stimuli relevant to understanding agents and their interactions (Happé et al., 2017). One of the main components of social cognition is theory of mind (ToM), identified as the ability that allows humans to explain and predict the behavior of others through the attribution of mental states to themselves and to others (Premack and Woodruff, 1978; Wimmer and Perner, 1983). Behavioral and neuroimaging studies identified two specific components of the ToM: an “affective” component that refers to the ability to infer the emotions felt by others, and a “cognitive” component that refers to the ability to make inferences about thoughts, beliefs, and intentions of other people (Shamay-Tsoory and Aharon-Peretz, 2007). Therefore, an efficient ToM is central to adequately act in the environment and to interact with others. ToM difficulties have been found in several neurodevelopmental conditions (Korkmaz, 2011) and in prematurely born children (Roldán-Tapia et al., 2017).

Moreover, deficits of ToM abilities have been found as a consequence of brain and peripheral nervous system injury in several clinical conditions and in elderly as a consequence of aging (e.g., Cho and Cohen, 2019; Wallis et al., 2022).

However, several questions remain open on the specific neural and neuropsychological correlates of the different components of ToM, and about the putative improvement of these abilities both in healthy participants and in patients affected by different neurological conditions. The goal of this Research Topic was to welcome the most recent and advanced research on ToM and social cognition, to better understand their characteristics and their recovery possibilities in different populations.

## Assessment of theory of mind and gender differences

To propose a new tool to evaluate ToM as a multidimensional construct, Isernia et al. adapted the Edinburgh Social Cognition Test (ESCoT) for the Italian healthy population. The ESCoT is a social cognition measure that uses animations of everyday social interactions to assess (i) cognitive theory of mind, (ii) affective theory of mind, (iii) social norm understanding (Baksh et al., 2018). They also investigated possible predictors of ESCoT performance, including demographic characteristics, IQ, and executive functions, to detect possible confounding variables. They found a good reliability of the ESCoT and an association with some traditional social cognition tests. Moreover, the ESCoT was associated with age and education, but not with general cognitive functioning.

The characterization of social functioning in healthy participants was also investigated by Fabbri et al. by testing whether the gender composition of interacting pairs modulated the joint action effect. Authors assessed how gender information of co-actors influenced the joint selective attention measured in a joint flanker and joint Navon tasks (see Atmaca et al., 2011; Böckler et al., 2012; Fabbri et al., 2018) by performing two experiments in neurotypical university students. Their findings showed that the gender composition of interacting pairs plays a role in joint attentional tasks.

## Difficulties and enhancement of theory of mind abilities

García et al. present a study aimed at analyzing social cognition performance in a sample of children with pediatric muscular dystrophies (i.e., a heterogeneous group of rare neuromuscular diseases characterized by progressive muscle degeneration) compared with a paired sample of healthy subjects, controlling for the effect of the general intelligence level and behavioral and emotional symptoms. They showed that children with pediatric muscular dystrophies performed significantly worse on most of social cognition tasks compared to healthy participants. In particular, both emotion recognition and ToM were impaired in in pediatric patients with muscular dystrophies. These findings highlight the importance to promote early interventions on social cognition.

On this subject, Bianco and Castelli performed a mini review of the current literature on the training programs developed to enhance key aspects of mature ToM skills. In detail, they examined results on trainings on second-order false belief reasoning, the ability to put one's own ToM knowledge into use, and the mentalization of thoughts and emotions. They also explored effects of these trainings on metacognition, social experience, and emotional competence.

Finally, d'Arma et al. propose a training protocol to enhance ToM abilities in in Multiple Sclerosis (MS), a neurodegenerative disease affecting young adults. They implemented an ecological training aimed composed by different activities (i.e., reading of stories, listening to audio voices, seeing a video-tape depicting human social interactions) to work on ToM capacity from different perspectives.

Results showed a significant enhancement of ToM after 3 weeks of training, in both affective and cognitive components, with a possible positive effect of patient's Quality of Life (QoL).

## Conclusions

In these Research Topic authors provided new evidence on social functioning in healthy subjects and propose a new tool for assessing ToM. Furthermore, they analyzed social cognition performance in a sample of children with pediatric muscular dystrophies. Finally, new insights into the enhancement of ToM abilities are proposed. Taken together, these findings represent an important contribution regarding social functioning and ToM in both healthy participants and clinical populations. Clearly, further studies are needed to better understand the complexity of these multidimensional constructs by focusing, for example, on the implementation of artificial intelligence in the field of ToM and its potential impact in rehabilitation research.

## Author contributions

AD: Conceptualization, Supervision, Writing—original draft, Writing—review & editing. CB: Writing—original draft, Writing—review & editing. MDRT: Writing—original draft, Writing—review & editing. GS: Writing— original draft, Writing—review & editing.
